# Silver Nanoparticles (AgNPs) in Urea Solution in Laboratory Tests and Field Experiments with Crops and Vegetables

**DOI:** 10.3390/ma15030870

**Published:** 2022-01-24

**Authors:** Dariusz Jaskulski, Iwona Jaskulska, Joanna Majewska, Maja Radziemska, Ayla Bilgin, Martin Brtnicky

**Affiliations:** 1 Department of Agronomy, Faculty of Agriculture and Biotechnology, Bydgoszcz University of Science and Technology, 7 Prof. S. Kaliskiego St., 85-796 Bydgoszcz, Poland; jaskulska@pbs.edu.pl; 2 Research & Development Centre Agro-Land Marek Różniak Śmielin, 89-110 Sadki, Poland; 3 Reckitt Banckiser Production Sp. z o.o., 05-100 Nowy Dwór Mazowiecki, Poland; joanna.majewska@rb.com; 4 Institute of Environmental Engineering, Warsaw University of Life Sciences, Nowoursynowska 159, 02-776 Warsaw, Poland; maja_radziemska@sggw.edu.pl; 5 Department of Agrochemistry, Soil Science, Microbiology and Plant Nutrition, Faculty of Agrisciences, Mendel University in Brno, Zemedelska 1, 61300 Brno, Czech Republic; martin.brtnicky@seznam.cz; 6 Faculty of Engineering, Artvin Coruh University, Seyitler Campus, Artvin 08000, Turkey; ayla.bilgin@artvin.edu.tr; 7 Institute of Chemistry and Technology of Environmental Protection, Faculty of Chemistry, Brno University of Technology, Purkynova 118, 61200 Brno, Czech Republic

**Keywords:** nanomaterials, nanotechnology, nanosilver, seed germination, plant growth, yields

## Abstract

Nanotechnology and nanomaterials, including silver nanoparticles (AgNPs), are increasingly important in modern science, economics, and agriculture. Their biological activity involves influencing plant health, physiological processes, growth, and yields, although they can also be toxic in the environment. A new fertiliser was made based on a urea solution with a relatively low content of AgNPs obtained by the reduction of silver nitrate V. Laboratory tests were used to assess the effect of a fertiliser solution containing 10 ppm AgNPs on the germination of agricultural plant seeds (barley, peas, oilseed rape) and vegetables (radish, cucumber, lettuce) and its foliar application on chlorophyll content, stomatal conductance, and seedling biomass. Field experiments were conducted to assess the effect that a foliar application of 15 ppm AgNPs in working liquid had on physiological plant parameters and yields of rape and cucumber. The AgNPs in the tested fertiliser reduced infestation of the germinating seeds by pathogens and positively affected the physiological processes, productivity, and yields of plants. Plant response depended on plant species and habitat conditions. Reduced pathogen infestation of seeds, higher germination energy, increased chlorophyll content and stomatal conductance, and higher seedling masses all occurred under the influence of AgNPs, mainly in oilseed rape and cucumber, and especially under thermal stress. The beneficial effect of AgNPs on the yield of these plants occurred in years of unfavourable weather conditions. The positive agricultural test results, especially under stress conditions, indicate that fertiliser produced with AgNPs as an ingredient may reduce the use of pesticides and highly concentrated mineral fertilisers. Such a fertiliser is fully in line with the idea of sustainable agriculture. However, research on the effects that AgNPs and fertiliser have on the environment and humans should continue.

## 1. Introduction

Nanotechnology refers to methods for obtaining objects of nano size, i.e., in at least one dimension measuring 1–100 nm [1]. Nanotechnology has progressed very rapidly in the latter 20th century and 21st century. However, there were successful attempts to obtain nanoparticles (NPs), including of silver, in the 18th and 19th centuries [2,3]. Studies on nanomaterials are of interest within many scientific fields, and their results are quickly being absorbed and implemented on the market. Nanomaterials are now widely used, e.g., in electronics and energy, medicine and pharmacy, cosmetics and environmental engineering, but also in agriculture. This is confirmed by numerous scientific reviews [4,5,6,7]. Used in medicine and cosmetology, nanotechnology can save human lives and improve quality of life. It facilitates diagnostics, the synthesis of active substances, and the transport of drugs in the body [8]. This field enables the development of space technologies and of common technologies of social value such as construction. Nanomaterials are increasingly present in electronic and electro-optical devices [9,10].

No less important and promising is the role of nanotechnology and nanomaterials in the development of agriculture and food science [11]. Manjunatha et al. [12] and Chhipa [13] indicate that agriculture is already benefitting from nanotechnology in biotechnology and hormone management, seed production, plant nutrition and protection, water management, biosensors, ecological and precision farming, and recycling of agricultural waste. Nanotechnology enables the assessment and improvement of environmental conditions, including via the purification and treatment of irrigation water and via increased soil water retention. The physicochemical properties of NPs result from their very small size and, thus, relatively large surface area, making them chemically and biologically active. Such properties of NPs make them increasingly used in agriculture [14,15]. They are used as biosensors, for transporting nutrients and pesticides in plants, for stimulating the growth and development of plants, and for increasing plant resistance to abiotic and biotic stress [16,17]. NPs can also be a source of plant nutrients. Nanofertilisers [18,19] are more effective than traditional mineral fertilisers containing macronutrients and/or microelements, such as salts, while also being environmentally safer. These are fertilisers with high nutrient bioavailabilities, but slow, prolonged action and controlled release into the environment. Nanoparticulate components of fertilisers or plant growth biostimulants can penetrate cells and even their organelles to affect the activity of enzymes as well as biochemical and physiological plant processes. In biological tests, the presence of NPs such as Al_2_O_3_, Au, C, Cu, Fe, Pb, Si, SiO_2_, TiO_2_, and ZnO has produced changes in, for example, the capacity and rate of seed germination; plant sprout, root, and shoot lengths; biomass production; and plant yields. There can also be changes in the parameters and effects of physiological processes, e.g., respiration, transpiration, photosynthesis, chlorophyll content, and productivity. These changes can be either favourable or unfavourable [20,21,22]. 

Of increasing importance in agriculture are silver nanoparticles (AgNPs), and the method for obtaining them (and their aseptic properties, which have been used in, for example, medicine) has been known for over 100 years [23,24,25,26]. In agriculture, AgNPs have been used in, among other things, the production of nanopesticides (mainly bactericides and fungicides), growth promoters, and fruit-ripening agents [27,28]. Agricultural studies confirm the high potential of AgNPs in reducing microbes that cause plant diseases and soil pathogens. However, the frequent use of AgNPs may lead to the development of resistance in microorganisms [29]. In addition to their bactericidal and fungicidal activity, AgNPs can also directly affect plant growth and development processes, just as fertilisers or biostimulants do. The positive and negative effects alike of AgNPs on the growth of crops—from germination, through physiological processes to conditioning yield size and quality—have been well documented in the scientific literature [30,31]. 

Nonetheless, AgNPs, similarly to other heavily and excessively often used NPs, may be toxic to organisms, contaminating water, soil, and air. Such an impact on environmental features depends on the properties of the NPs—on their size, shape, and specific surface—and on environmental conditions [32,33]. Therefore, permanent research in this area is needed. Decision makers in science and the economy alike agree that nanotechnology will be an important part of mankind’s civilisational development, including in agriculture and food production. Future work should focus on methods for obtaining and using NPs, particularly on solutions to reduce the risk of their harmful human and environmental effects [34]. Therefore, it was assumed that it is possible to obtain a fertiliser solution with AgNPs based on urea (which is widely used in agriculture) that will positively affect the germination of seeds of agricultural and horticultural plants and stimulate their growth and, consequently, increase yields. Therefore, the research objective was to verify this hypothesis via a series of laboratory tests and field experiments.

## 2. Materials and Methods

The research was carried out by an international team of specialists in the fields of agriculture and environmental protection. The laboratory tests and field experiments were done at the Research & Development Centre Agro-Land Marek Różniak Śmielin (53°09′04.0″ N; 17°29′10.7″ E) in the Kujawsko-Pomorskie Voivodeship, Poland and at the Department of Agronomy of the Bydgoszcz University of Science and Technology in Bydgoszcz, Poland. 

### 2.1. Fertiliser Containing AgNPs 

**Figure 1:**
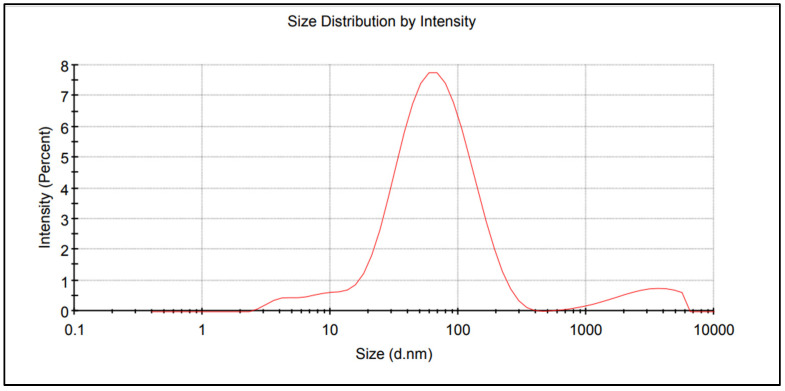
Size distribution report of AgNPs.

The nitrogen fertiliser with AgNPs was obtained in three stages. In the first, a 15% *m*/*m* solution of urea was produced in demineralised water of low electrolytic conductivity (0.5–2.0 μs/cm) and pH of 5 to 7. The second stage involved the preparation of AgNPs by the chemical reduction of silver nitrate V in an aqueous solution of ammonium nitrate V in the presence of PVP (polyvinylpyrrolidone—non-toxic NPs stabilizer) and a non-ionic surfactant for the stabilisation and improved dispersion of AgNPs. The resulting AgNPs precipitate was separated from the solution by centrifugation. The evaluation and characterization of NPs can be performed using various techniques, including UV-Vis spectrum, Transmission Electron Microscope (TEM), X-ray diffraction (XRD), and Scanning Electron Microscopy (SEM). The authors had the opportunity to use the method of measuring the size of NPs dispersed in a liquid—Dynamic Light Scattering (DLS). The AgNPs distribution report made using the Zetasizer analyser (Malvern Instruments Ltd. Worcestershire, WR14 1XZ, UK) is shown in Figure 1. In the third stage, the AgNP precipitate was distributed into the urea solution prepared in the first stage in an amount appropriate to the laboratory test and field experiment assumptions. 

### 2.2. Laboratory Tests

The laboratory tests (series I) assessed the effect of AgNPs on seed germination and the initial growth of three types of agricultural crops (barley, peas, oilseed rape) and three species of vegetables (radish, cucumber, lettuce). Seed germination was performed in accordance with principles established by the International Seed Testing Association (ISTA) [35] for optimal conditions; and in line with the methodology, but at a 5 °C lower temperature for stress conditions. The experimental factor was the medium used to moisten the seed germination substrate. The substrate was moistened with urea solution containing 10 ppm AgNPs (AgNPs + Urea); urea solution as control I (Urea); and demineralised water as control II (Water). The characteristics assessed for germinating seeds were germination energy, germination capacity, number of abnormal sprouts per 100 sprouted seeds, sprout length, and fungal infection of germinating seeds. 

In a vegetation chamber capable of regulating plant growth conditions (series II), the effect of foliar application of AgNPs on the content of chlorophyll, stomatal conductance, and plant biomass in the 4–6 leaf phase was assessed. Plant growth tests were performed in optimal habitat conditions. The lengths of day and night were 16 and 8 h, respectively. The air temperature was 20 °C during the day and 13 °C at night, except for cucumber growth (24 °C and 18 °C, respectively). The plants were also subjected to environmental stress. Thermal stress consisted in lowering the temperature by 5 °C for day and 3 °C for night, and water stress in reducing the water capacity of the substrate from 60% to 40% field capacity. Chlorophyll content was determined using a CM1000 type non-contact chlorophyll meter (Spectrum Technologies, Inc. Thayer Court, Aurora, IL, USA). Stomatal conductance was measured with an SC-1 Leaf Porometer (METER Group, Inc., Pullman, WA, USA). At the end of the research period, biomass (dry weight of plants) was determined by dry-weight at 105 °C using a Solid Line FD-S 115 drier with forced air circulation (BINDER GmbH, Tuttlingen, Germany) and a laboratory balance. 

### 2.3. Field Experiments

In the years 2019–2021, two field experiments were carried out, one on an agricultural crop (winter oilseed rape) and the other on a vegetable plant (cucumber). Plant species were selected based on the results of laboratory tests in which plants reacted significantly to the presence of AgNPs with a change in size of germination and initial growth parameters, especially in comparison with the effect of urea solution. Cucumber was tested for three years as a spring plant, and oilseed rape was tested for two full vegetation seasons as a winter plant. The field experiments were carried out on sandy loam Luvisol [36]. 

The research area is located in a humid continental climate zone. According to Köppen [37], it is classified as Dfb climate (cold, without dry season, warm summer). Soil properties and meteorological conditions at the site and during the study period are presented in Table 1 and Table 2.

The experimental factor in both experiments was the foliar application of AgNPs. As in the laboratory tests, three variants were used: a fertiliser consisting of urea solution with AgNPs (AgNPs + Urea) and two controls—urea solution (Urea) and water (Water). As the working liquid for foliar application, 200 L·ha^−1^ was prepared based on water with the addition of, respectively, 15 ppm AgNPs + 37.5 g of urea; 0.0 ppm AgNPs + 37.5 g of urea; and 0.0 ppm AgNPs + 0.0 g of urea. In the oilseed rape cultivation, foliar treatments were performed three times during the growing season: first in autumn in the BBCH 16 phase and twice in the spring at BBCH 21 and BBCH 51. Two treatments were applied to cucumber: at BBCH 25 and BBCH 51. The physiological parameters of plants of both species—leaf cover index (LAI), photosynthetically active radiation (PAR), and chlorophyll content in leaves—were determined at the onset of flowering (BBCH 61) and in rape in 2019 after the study had begun only in autumn (BBCH 18–20). Measurements were made using the equipment used in the laboratory tests. PAR was determined using an AccuPAR LP−80 PAR/LAI meter (METER Group, Inc.). Intercepted photosynthetically active radiation (IPAR%) was calculated using PAR measurements above the plant canopy and in the canopy at the soil surface level. The measure of plant productivity was yield, i.e., mass of rape seeds and cucumber fruits expressed in tonnes per hectare.

### 2.4. Analysis of Data

The data from biometric and physiological measurements were subjected to statistical analysis. Individual datasets were assumed to be normally distributed, and this was verified using the Shapiro–Wilk test. Data that was not normally distributed were transformed. The percentage results (germination energy, germination capacity) were transformed according to the Bliss rule (arc sin). ANOVA was performed. The statistical significance of the influence of experimental treatments was assessed by the F test, and the significance of differences between mean values of individual characteristics was assessed by Tukey’s post hoc test at *p* < 0.05. In the tables of results and on the figures, the letters a, b, and c indicate significant differences in the values of plant features under the influence of the investigated treatments. Faced with the impact of experimental treatments on physiological parameters and crop yields differing significantly between successive years of field experiments, the results for each year of the study have been presented separately. The results were processed mathematically and statistically in Statistica.PL 12 [38].

## 3. Results

Under habitat conditions optimal for germinating crop seeds, adding AgNPs to the substrate did not significantly affect germination energy, germination capacity, sprout length, or the number of abnormal sprouts for barley, peas, and rape. Only the length of the rapeseed sprout was greater in the presence of AgNPs with urea than in the substrate moistened only with water. However, AgNPs decreased the infection of pea seeds with pathogens as compared to their infection on a substrate moistened with urea solution and clean water (Table 3). Under thermal stress, the presence of AgNPs in the substrate increased the germination energy and length of barley sprout in comparison with the seed germinated on a substrate moistened with clean water. The germination energy and length of rapeseed sprout were greater under the influence of AgNPs than on the substrate treated with urea solution and water, and the germination capacity was greater than on the substrate moistened with water. AgNPs also reduced the pathogen contamination of germinating seeds of the tested plants under thermal stress conditions.

Under thermal stress conditions, AgNPs in urea solution significantly increased the germination energy of cucumber seeds in comparison with germination on the control substrate treated with urea solution and water, and of radish seeds in comparison to the substrate moistened with water. The germination energy of cucumber seeds was higher under the influence of AgNPs than on the substrate moistened with water, including under optimal thermal conditions (Table 4). AgNPs had a beneficial effect on seed germination under stress conditions only—for radish compared to sprouting in the water-moistened substrate and for cucumber compared to germination on both control substrates. Under stress conditions, as compared to germination on both control substrates, the presence of AgNPs in the substrate increased the sprout length and decreased the number of abnormal sprouts for cucumber, as well as reducing pathogen infestation for all vegetable species. For cucumber and lettuce, the infestation of sprouts was reduced under the influence of AgNPs under optimal conditions, too.

**Figure 2:**
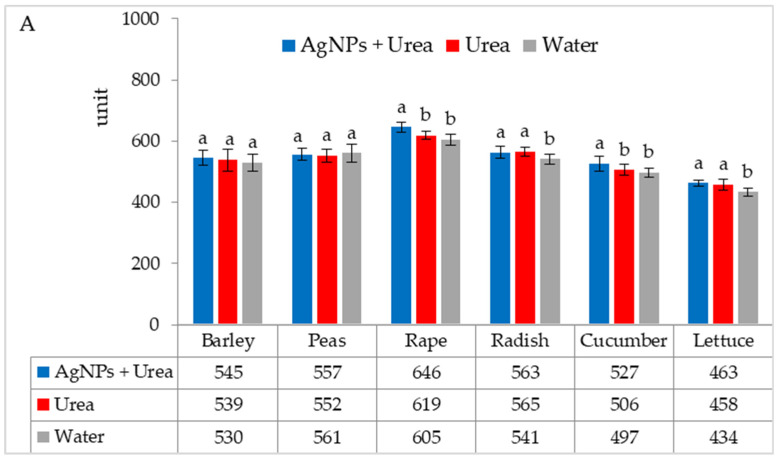

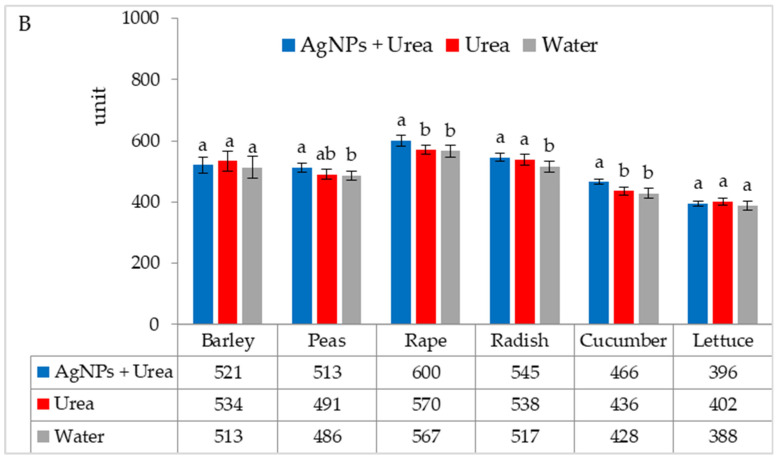
Chlorophyll content in leaves under optimal—(**A**) and thermal stress conditions—(**B**) depending on the presence of AgNPs in the substrate; urea solution with AgNPs (AgNPs + Urea), urea solution (Urea), water (Water). a, b—letters indicate a significant difference in the content of chlorophyll for individual plant species at *p* < 0.05.

In laboratory tests, under optimal thermal conditions, chlorophyll contents were significantly higher in rape and cucumber leaves treated with urea solution with AgNPs than in plants treated with urea solution and water. On the other hand, the content of chlorophyll in radish and lettuce leaves was higher under the influence of urea solution with AgNPs than in control plants sprayed with pure water only (Figure 2A). The foliar application of urea + AgNPs solution under thermal stress significantly increased the chlorophyll content in rape and cucumber leaves, as well as in peas and radishes, but only in comparison with plants treated with water (Figure 2B).

**Figure 3:**
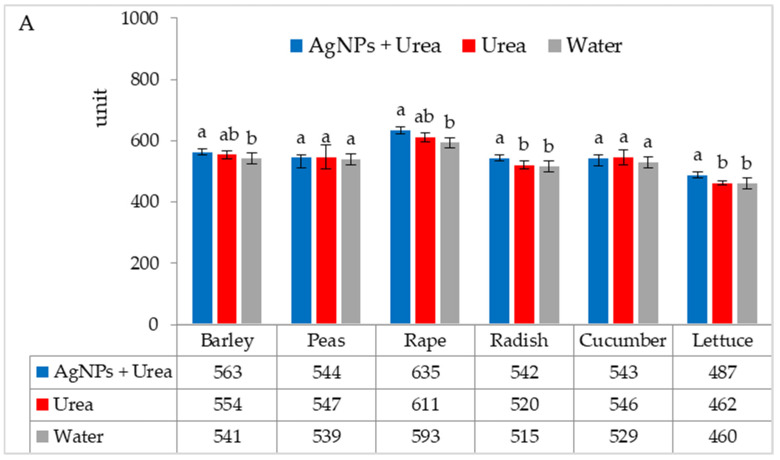

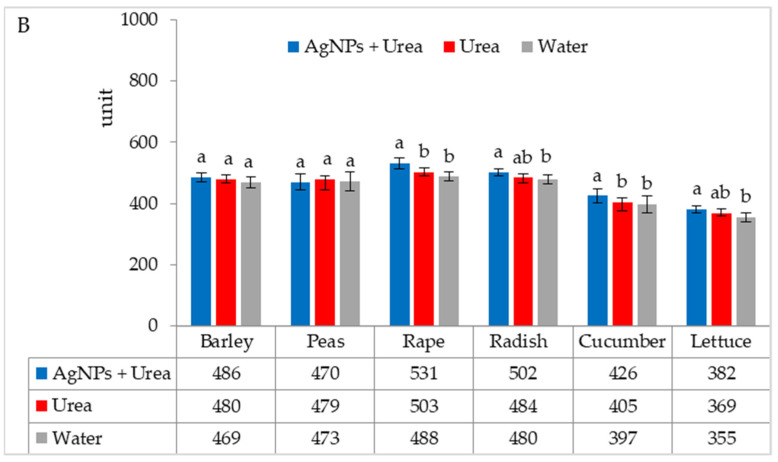
Chlorophyll content in leaves under optimal—(**A**) and water stress conditions—(**B**) depending on the presence of AgNPs in the substrate; urea solution with AgNPs (AgNPs + Urea), urea solution (Urea), water (Water). a, b—letters indicate a significant difference in the content of chlorophyll for individual plant species at *p* < 0.05.

Chlorophyll content in leaves under optimal substrate water conditions was higher after foliar application of urea solution + AgNPs than after exposure to water (for barley and rape) and after application of both urea solution and water (for radish and lettuce) (Figure 3A). Under water stress conditions, the AgNPs in urea solution significantly increased the chlorophyll content in rape and cucumber leaves as compared to urea solution alone and as compared to pure water for leaves of radish and lettuce (Figure 3B).

**Figure 4:**
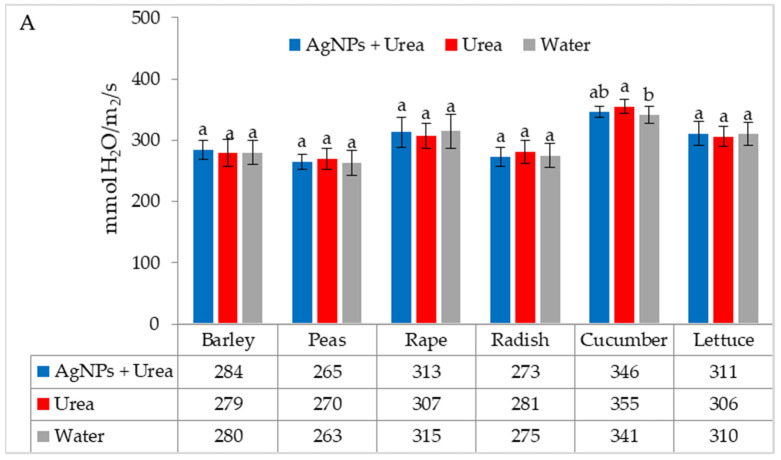

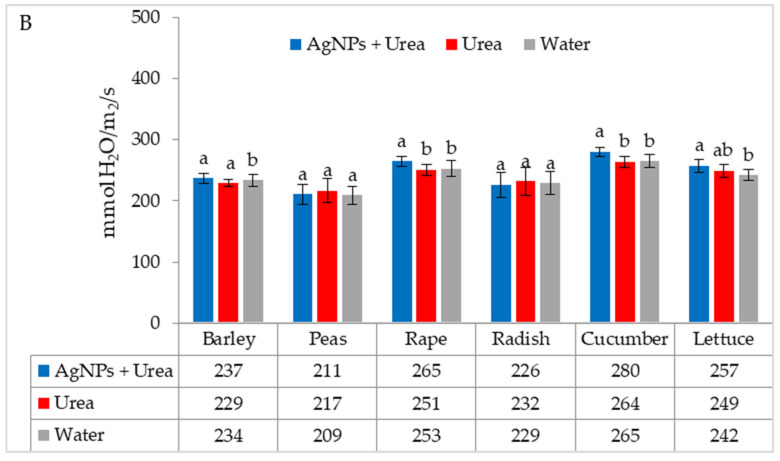
The leaf stomatal conductance of plants under optimal—(**A**) and thermal stress conditions—(**B**) depending on the presence of AgNPs in the substrate; urea solution with AgNPs (AgNPs + Urea), urea solution (Urea), water (Water). a, b—letters indicate a significant difference in the stomatal conductivity for individual plant species at *p* < 0.05.

Under optimal thermal conditions, the foliar application of AgNPs in urea solution and in urea and water produced a different stomatal conductance in cucumber alone. This characteristic did not differ significantly under the influence of urea and urea solution with AgNPs, although it was greater with the application of urea solution than under the influence of water (Figure 4A). Under thermal stress, the application of AgNPs in urea solution increased stomatal conductance in comparison to spraying with urea solution and water (for rape and cucumber) or with only water (for barley and lettuce) (Figure 4B).

**Figure 5:**
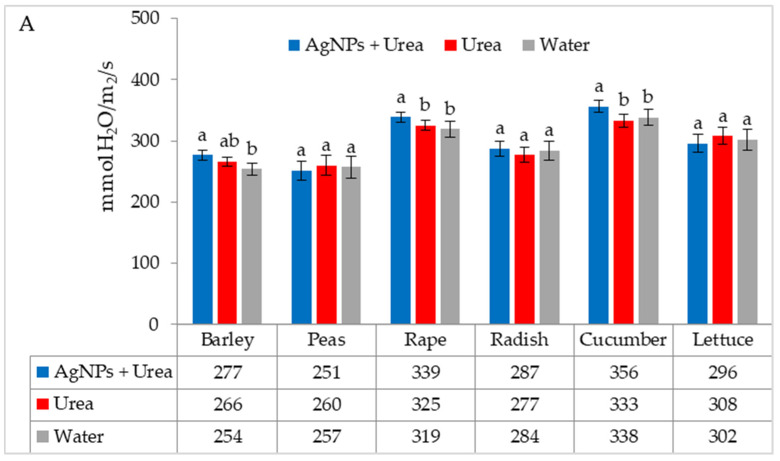

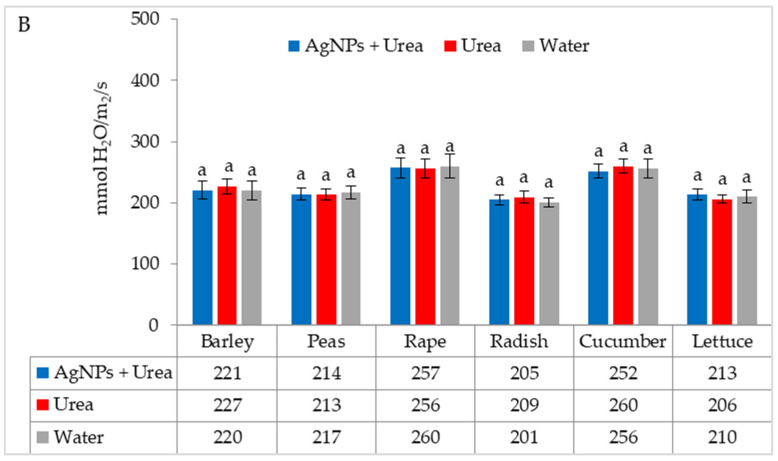
The leaf stomatal conductance of plants under optimal—(**A**) and water stress conditions—(**B**) depending on the presence of AgNPs in the substrate; urea solution with AgNPs (AgNPs + Urea), urea solution (Urea), water (Water). a, b—letters indicate a significant difference in the stomatal conductivity for individual plant species at *p* < 0.05.

The stomatal conductance of rape and cucumber was increased under optimal water conditions by the application of AgNPs. This physiological parameter was also significantly higher in barley fertilised with urea + AgNPs solution but only as compared to plants treated with water (Figure 5A). However, AgNPs did not affect the stomatal conductance of seedlings of plants growing under water stress (Figure 5B).

**Figure 6:**
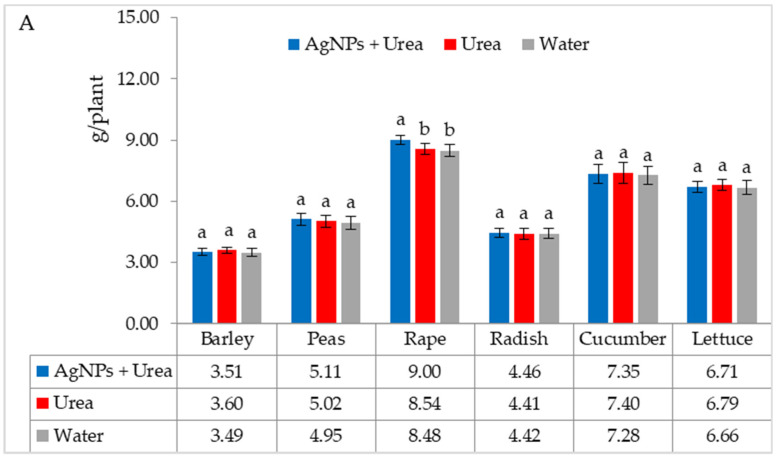

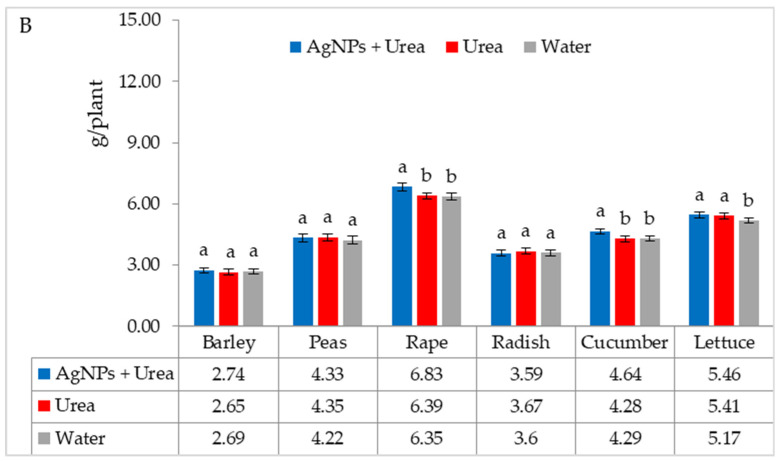
Plant biomass under optimal—(**A**) and thermal stress conditions—(**B**) depending on the presence of AgNPs in the substrate; urea solution with AgNPs (AgNPs + Urea), urea solution (Urea), water (Water). a, b—letters indicate a significant difference in the biomass of individual plant species at *p* < 0.05.

The mass of seedlings of plants growing in optimal thermal conditions was not significantly affected by the foliar application of AgNPs. The exception was the reaction of rape, whose plant mass was 6.1% than when treated with water (Figure 6A). On the other hand, under thermal stress, the mass of rape and cucumber seedlings was 6.9% and 8.4% higher, respectively, than plants treated with urea solution, and 7.5% and 8.1% higher than plants treated with water. Under these conditions, the mass of lettuce seedlings under the influence of AgNPs was also higher, but only in comparison with plants treated with water. The relative difference in plant mass was 5.7% (Figure 6B).

**Figure 7:**
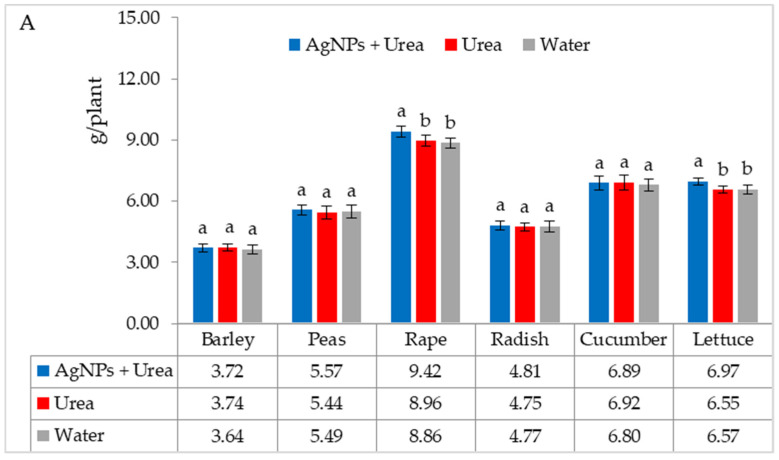

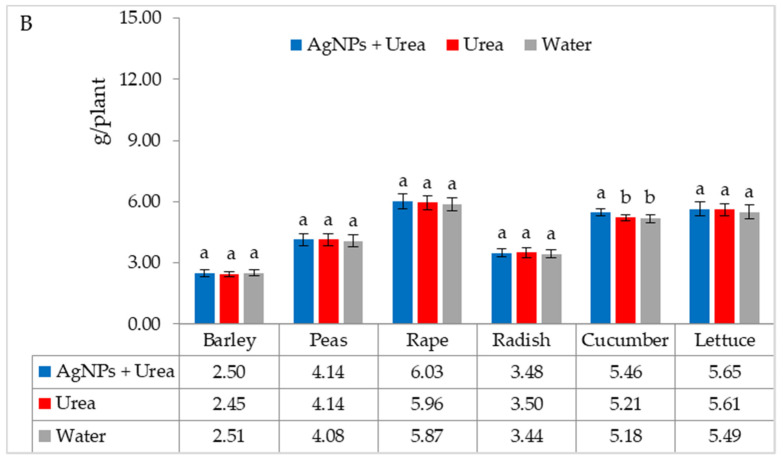
Plant biomass under optimal—(**A**) and water stress conditions—(**B**) depending on the presence of AgNPs in the substrate; urea solution with AgNPs (AgNPs + Urea), urea solution (Urea), water (Water). a, b—letters indicate a significant difference in the biomass of individual plant species at *p* < 0.05.

Under optimal water conditions, the mass of rape seedlings was 6.3% higher under the influence of AgNPs than treatment with water. The same response in lettuce was 6.1% (Figure 7A). However, under water stress, there was no significant effect of AgNPs on the mass of plant seedlings with the exception of cucumber. AgNPs used with urea solution increased the mass of cucumber seedlings by 5.5% compared to the mass of plants treated with water (Figure 7B).

The effect of AgNPs on the physiological canopy parameters differed between study years and between plant species in field experiments (Table 5). The foliar application of AgNPs significantly increased the LAI index of rape in 2019 but only relative to plants sprayed with water. The LAI index for cucumber in 2019 and 2021 was significantly higher under the influence of AgNPs than without treatment with nanoparticles. There was a beneficial effect of AgNPs on the IPAR index for rape in 2019 and 2020, and for cucumber in 2019 and 2021, but only compared to plants sprayed with water. The chlorophyll content in rape leaves was higher after the application of AgNPs than after the application of urea and water solution in 2019 and 2021. Cucumber leaves contained more chlorophylls after AgNPs application than after water treatment of plants in 2019 only.

**Figure 8:**
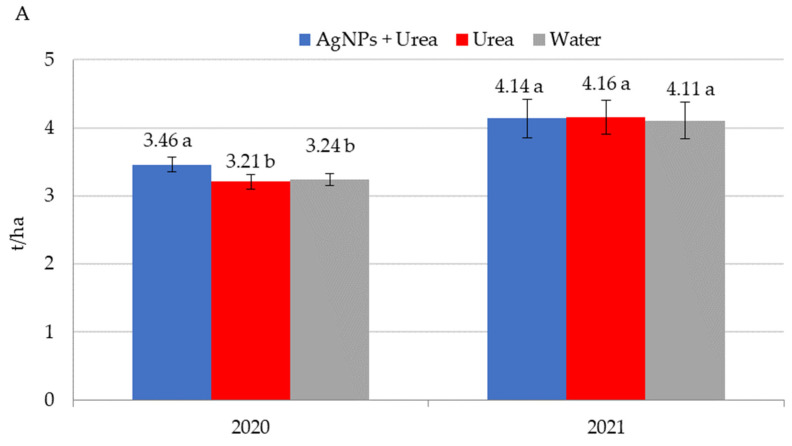

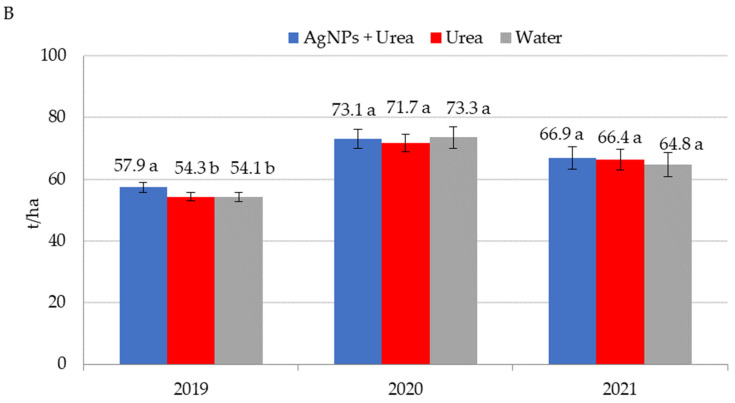
Rape—(**A**) and cucumber yield—(**B**) depending on the foliar application of AgNPs treatments; urea solution with AgNPs (AgNPs + Urea), urea solution (Urea), water (Water). a, b—letters indicate a significant difference in yields of individual plant species at *p* < 0.05.

Application of AgNPs three times during the growing season of rape increased seed yields in 2020 (Figure 8A). The increased yield was 0.25 t·ha^−1^ (i.e., 7.8%) over control plants sprayed with urea solution and 0.22 t·ha^−1^ (i.e., 6.8%) over control plants treated with water. In 2021, foliar treatments, including those involving AgNPs, did not significantly differentiate seed yields for rape. AgNPs had a beneficial effect on cucumber yield in 2019, but there was no such significant effect in 2020 and 2021 (Figure 8B). In 2019, cucumber yield was 3.6–3.8 t·ha^−1^ higher under the influence of AgNPs than in control plants (treated with urea solution and water). Therefore, the difference was 6.6–7.0%. In 2021, the trend of increased yields relative to control plants treated with water, although statistically insignificant, amounted to 3.2%.

## 4. Discussion

The results of the laboratory tests and field experiments confirm AgNPs’ potential impact on crops, as presented in the literature [39,40,41]. However, in the cited studies, the concentration of NPs in the plant growth environment was often many times higher than in our studies, amounting to even 500–1000 ppm [42,43]. This impact resulted both in favourable plant reactions such as improved germination, growth, and yield, and in unfavourable changes, including plant death [44,45,46]. In the author’s own research presented in this study, the working liquid for treating plants contained 10–15 ppm of AgNPs. More than 90% of the AgNPs had a size of 1–100 nm. The average size of AgNPs was 48 nm. Such NPs are more environmentally safe than smaller sized NPs. Sukhanova et al. [47] and Egbuna et al. [48] indicate that the biological activity and toxicity of NPs, including silver, are the greater the smaller the size of the NPs. In addition, the methodological assumptions and experiment design allowed the conclusion to be supported that the observed plant reactions result from the impact of AgNPs and not of other components in the solutions applied to the seed germination substrate or in the foliar treatments. That is why two control objects were used in these studies (urea solution and water). This allowed the possible effect on plants of the nitrogen in urea to be eliminated in the results. Hence, it was used in the technology for producing and fixing AgNPs, although its concentration in the solutions used to moisten the substrate or foliar application was very low, not exceeding 0.04% N. 

The disinfectant action of AgNPs, which is known and applied on a larger scale in medicine [49,50], has been investigated in recent decades for possible application in protecting agricultural and horticultural plants [51,52]. The AgNPs used in our research significantly reduced the infestation of germinating seeds by pathogens. This was especially noticeable at lower temperatures, which prolonged the germination period of seeds and, thus, also the pressure of pathogens. Therefore, the potential to use the produced AgNPs to disinfect seeds without using pesticides has been proven. Importantly, this effect was achieved despite the relatively low concentration of AgNPs in the substrate. Wolny-Koładka et al. [53] showed that the fungicidal activity of AgNPs obtained by chemical reduction against fungi of the species *Fusarium culmorum* occurred at a much higher concentration, i.e., 60 ppm. However, the scientific literature contains results confirming the fungicidal activity of AgNPs at relatively low NPs concentrations. The growth of *Fusarium* spp. was inhibited by AgNPs already at a concentration of 2.5 ppm according to Kasprowicz et al. [54] and at 10 ppm according to Kim et al. [55]. The biological activity of low concentrations of AgNPs is of economic and environmental importance. The frequent use of large amounts of AgNPs may be environmentally toxic and lead to the development of resistance in microorganisms [56,57]. 

Another important agricultural use of AgNPs, apart from in plant protection, is in biostimulation. The complex nature of NPs’ influence on plants results from their ability to penetrate cells and on into subcellular organelles such as nuclei, plastids, and vacuoles [58,59]. Under the influence of NPs, changes have been seen in, for example, chlorophyll content and photosynthesis rate, stomatal conductance, intercellular CO_2_ concentration, and the synthesis and activity of some enzymes [60,61]. Changes at the DNA level are also possible [62]. Depending on the type, shape, size, and concentration of NPs, plant responses to their presence may vary, from stimulation of growth and development, through inhibition, to die-off [63]. The results of studies on agricultural and horticultural plants indicate that these effects also occur under the influence of AgNPs. Szőllősi et al. [64], based on a review of the results of numerous studies on the impact of nanomaterials on the germination and growth of seedlings, divided plant reactions to AgNPs into positive, negative, and neutral (no significant reaction). The judicious use of AgNPs can increase and improve seed germination, root length and mass, number of leaves, plant size and mass, and seed yield [65,66,67]. The application of appropriate concentrations of AgNPs also allows the content of plant pigments and enzymatic activity to be increased [68,69]. Nonetheless, plant reactions are specific and species-dependent. In our own research, 10 ppm of AgNP concentration in the solution used to moisten the substrate significantly improved the germination energy of rape and cucumber seeds, but it did not significantly affect the germination of barley, pea, radish, or lettuce. The positive response of rape and cucumber to AgNPs was also reflected in longer sprouts, higher chlorophyll content, and higher stomatal conductance, especially when young plants were grown under thermal stress. Such results are partially confirmed by other authors’ studies on various plant species, including those of the *Cucurbitaceae* family. Almutairi and Alharbi [70] found an improvement in the germination of *Cucurbita pepo* L. seeds under the influence of AgNPs in concentrations of 0.5–2.0 mg·L^−1^, with no such effect on lettuce seeds. Barrena et al. [71], too, found no (positive or negative) effect of AgNPs at a concentration of 100 µg mL^−1^ on the germination index of lettuce seeds. 

In the field experiments, the response of plants to foliar application of AgNPs depended on environmental conditions (study year) and plant species (rape, cucumber). Depending on the year of research (the years having been characterised by different meteorological conditions, see Table 2), the degree of positive reaction of rapeseed and cucumber to AgNPs varied. This was reflected in a significant differentiation in chlorophyll content in leaves as well as the LAI and IPAR indices. Yields increased significantly due to the foliar application of AgNPs for rape in 2020 and cucumber in 2019. These were the years of lowest yields, as resulted from stressful habitat (weather) conditions. In 2019, during the intensive growth and yielding of cucumbers in June–August, the sum of monthly rainfall was much below average for the study area, while the average air temperatures were above average. On the other hand, in 2020, while rape yield was being built up, rainfall was below the norm in April and May but three times higher than average in June. These results confirm the conclusion drawn from the studies by Prażak et al. [72] that AgNPs activate plant mechanisms, increasing tolerance to environmental stress. The authors found that AgNP concentrations of 0.25–1.25 mg·L^−1^ increased the rate and uniformity of bean seed germination in laboratory and field tests performed under unfavourable thermal conditions. In the late vegetation period, these plants also had greater seedling heights and masses and greater net photosynthesis.

## 5. Conclusions

Under chemical laboratory conditions, it is possible to produce AgNPs and, thence, urea-based agricultural fertiliser containing them. Biological laboratory tests and field experiments demonstrated that at concentrations of 10–15 ppm, AgNPs thus obtained had a positive effect on the seed germination, physiological parameters, and productivity of agricultural and horticultural plants. However, the reaction of plants to AgNPs depends on their species and on environmental, water, thermal, and weather conditions. The demonstrated ability to reduce the occurrence of pathogens affecting germinating seed, and to stimulate physiological processes, plant productivity, and yields, especially under environmental stress conditions, leads to the assumption that AgNPs thus obtained may have a place in sustainable agriculture. Using AgNPs in a way that can bring to bear their beneficial effects on the health, growth, and yields of plants could reduce the use of chemical production resources such as fertilisers or pesticides. The positive results of initial agricultural tests do not negate the need for further research on the effects that AgNPs and fertilisers containing them have on elements of the environment, including animals and humans.

## Figures and Tables

**Table 1:** Soil properties in site of field experiments.

Property	2019	2020	2021
Rape			
Texture (%)			
Sand (2–0.05 mm)	49.5	53.6	-
Silt (0.05–0.002 mm)	45.2	44.1	-
Clay (<0.002 mm)	5.3	2.3	-
pHKCl	6.52	5.93	-
Organic carbon (g C kg^−1^ soil)	11.2	10.6	-
Total nitrogen (g N kg^−1^ soil)	1.07	0.95	-
Available nutrients			
Phosphorus (mg P kg^−1^ soil)	96.5	117.2	-
Potassium (mg K kg^−1^ soil)	162.1	146.8	-
Magnesium (mg kg^−1^ soil)	63.0	42.7	-
Cucumber			
Texture (%)			
Sand (2–0.05 mm)	45.1	46.3	44.7
Silt (0.05–0.002 mm)	48.3	46.9	49.6
Clay (<0.002 mm)	6.6	6.8	5.7
pHKCl	7.08	6.61	6.85
Organic carbon (g C kg^−1^ soil)	12.9	11.7	13.5
Total nitrogen (g N kg^−1^ soil)	1.13	1.20	1.42
Available nutrients			
Phosphorus (mg P kg^−1^ soil)	114.8	98.0	120.4
Potassium (mg K kg^−1^ soil)	188.9	177.3	203.5
Magnesium (mg kg^−1^ soil)	55.6	60.4	66.2

**Table 2:** Air temperature and precipitation during the field experiments period.

Year								
Month	2019	2020	2021	Many-Year	2019	2020	2021	Many-Year
Temperature (°C)					Precipitation (mm)			
January	-	2.6	−1.1	−1.8	-	37.7	28.3	26.8
February	-	3.6	−1.8	−0.9	-	36.0	0.8	20.7
March	-	3.9	3.7	2.5	-	26.1	21.7	31.9
April	-	8.2	6.2	7.9	-	0.7	30.7	27.0
May	-	11.2	12.2	13.3	-	34.2	75.2	49.3
June	-	17.9	20.1	16.1	-	142.0	30.1	52.8
July	-	18.3	20.9	18.6	-	67.2	61.7	69.8
August	19.7	19.9	17.4	17.9	37.7	114.4	38.1	62.6
September	13.5	15.1	-	13.1	98.5	66.7	-	46.0
October	9.8	10.5	-	8.2	35.9	72.9	-	31.5
November	5.5	6.0	-	2.9	69.6	12.4	-	32.4
December	2.7	1.8	-	−0.6	21.1	33.8	-	34.0

**Table 3:** Germination of crop seeds in the presence of AgNPs in the substrate; urea solution with AgNPs (AgNPs + Urea), urea solution (Urea), water (Water). a, b—letters in the rows for optimal and stress conditions indicate a significant difference between the values of a given feature of plants at *p* < 0.05.

Feature	AgNPs + Urea	Urea	Water	AgNPs + Urea	Urea	Water
	Optimal Conditions			Stress Conditions		
	Barley					
Germination energy, %	84.3 a (±5.2)	85.0 a (±4.9)	86.0 a (±4.4)	77.1 a (±1.9)	75.3 a,b (±1.4)	72.2 b (±2.3)
Germination capacity, %	93.2 a (±3.6)	92.5 a (±3.5)	93.1 a (±4.2)	90.2 a (±2.8)	86.9 a (±2.6)	90.4 a (±2.5)
Abnormal sprouts, pcs/100 seeds	2.4 a (±0.45)	2.1 a (±0.27)	2.3 a (±0.35)	2.1 a (±0.24)	2.2 a (±0.26)	2.2 a (±0.18)
Sprout length, mm	87.4 a (±4.9)	88.0 a (±4.3)	87.2 a (±5.7)	62.5 a (±1.5)	60.4 a,b (±2.3)	56.6 b (±2.3)
Pathogen infestation, %	5.3 a (±0.37)	5.7 a (±0.42)	5.5 a (±0.22)	5.1 b (±0.22)	9.0 a (±0.24)	8.8 a (±0.18)
	Peas					
Germination energy, %	76.7 a (±3.7)	78.0 a (±3.4)	77.3 a (±4.0)	66.2 a (±4.3)	64.6 a (±4.2)	64.2 a (±4.2)
Germination capacity, %	88.9 a (±4.8)	88.7 a (±3.7)	90.3 a (±4.0)	82.5 a (±6.1)	80.8 a (±4.1)	82.6 a (±5.0)
Abnormal sprouts, pcs/100 seeds	3.9 a (±0.29)	4.2 a (±0.36)	3.9 a (±0.32)	3.6 a (±0.38)	3.8 a (±0.43)	3.8 a (±0.29)
Sprout length, mm	53.1 a (±3.5)	54.0 a (±6.1)	52.8 a (±3.4)	45.6 a (±2.8)	44.5 a (±3.1)	44.4 a (±2.9)
Pathogen infestation, %	7.8 b (±0.53)	9.7 a (±0.67)	10.0 a (±0.78)	7.5 b (±0.49)	12.8 a (±1.33)	12.6 a (±0.90)
	Rape					
Germination energy, %	90.2 a (±4.2)	87.1 a (±4.4)	87.0 a (±5.4)	86.3 a (±2.3)	81.9 b (±1.1)	82.2 b (±1.4)
Germination capacity, %	95.0 a (±3.7)	96.1 a (±2.8)	94.9 a (±6.1)	91.7 a (±3.2)	87.2 a,b (±2.1)	86.4 b (±2.0)
Abnormal sprouts, pcs/100 seeds	2.7 a (±0.52)	2.6 a (±0.36)	3.1 a (±0.32)	3.3 a (±0.45)	2.9 a (±0.23)	3.3 a (±0.50)
Sprout length, mm	57.8 a (±1.2)	56.3 a,b (±1.6)	54.8 b (±1.5)	47.9 a (±1.6)	44.5 b (±1.5)	44.0 b (±1.7)
Pathogen infestation, %	12.7 a (±0.78)	13.4 a (±1.16)	13.0 a (±0.74)	12.4 b (±0.63)	18.5 a (±0.94)	18.1 a (±0.98)

**Table 4:** Germination of vegetable seeds in the presence of AgNPs in the substrate; urea solution with AgNPs (AgNPs + Urea), urea solution (Urea), water (Water). a, b—letters in the rows for optimal and stress conditions indicate a significant difference between the values of a given feature of plants at *p* < 0.05.

Feature	AgNPs + Urea	Urea	Water	AgNPs + Urea	Urea	Water
	Optimal Conditions			Stress Conditions		
	Radish					
Germination energy, %	82.5 a (±5.4)	84.0 a (±5.0)	83.4 a (±4.8)	74.8 a (±1.4)	73.3 a,b (±1.4)	71.1 b (±2.3)
Germination capacity, %	88.5 a (±4.1)	86.7 a (±3.7)	87.7 a (±4.6)	80.8 a (±2.5)	78.2 a (±2.0)	75.0 b (±2.5)
Abnormal sprouts, pcs/100 seeds	3.7 a (±0.41)	3.9 a (±0.29)	3.6 a (±0.29)	3.5 a (±0.41)	3.6 a (±0.24)	3.4 a (±0.22)
Sprout length, mm	46.7 a (±4.8)	44.7 a (±2.9)	45.0 a (±2.2)	41.9 a (±3.1)	38.9 a (±1.8)	39.6 a (±2.4)
Pathogen infestation, %	10.3 a (±1.48)	11.1 a (±0.82)	11.4 a (±1.00)	12.4 b (±1.25)	18.6 a (±1.39)	19.5 a (±1.90)
	Cucumber					
Germination energy, %	76.5 a (±1.6)	74.2 a,b (±1.4)	72.9 b (±1.8)	70.5 a (±1.4)	67.3 b (±1.4)	66.2 b (±1.8)
Germination capacity, %	85.3 a (±2.4)	84.7 a (±3.4)	83.0 a (±3.4)	81.7 a (±3.2)	75.3 b (±3.6)	76.4 b (±2.7)
Abnormal sprouts, pcs/100 seeds	4.1 a (±0.24)	4.3 a (±0.35)	4.0 a (±0.24)	4.3 b (±0.14)	4.7 a (±0.18)	4.7 a (±0.24)
Sprout length, mm	42.7 a (±3.3)	40.9 a (±2.2)	42.8 a (±3.2)	38.2 a (±2.0)	34.6 b (±1.9)	33.8 b (±1.4)
Pathogen infestation, %	8.7 b (±0.67)	17.2 a (±1.88)	16.6 a (±1.43)	7.0 b (±0.92)	15.5 a (±2.22)	16.2 a (±1.23)
	Lettuce					
Germination energy, %	64.6 a (±3.1)	65.0 a (±3.0)	65.9 a (±4.9)	59.8 a (±3.9)	57.7 a (±3.7)	58.6 a (±4.1)
Germination capacity, %	82.4 a (±3.5)	83.2 a (±4.0)	80.1 a (±3.2)	84.7 a (±2.7)	84.6 a (±2.7)	79.7 b (±2.5)
Abnormal sprouts, pcs/100 seeds	5.1 a (±0.32)	5.2 a (±0.38)	4.8 a (±0.24)	5.0 b (±0.26)	5.8 a (±0.24)	6.1 a (±0.36)
Sprout length, mm	48.8 a (±1.4)	47.1 a (±1.8)	45.2 b (±1.5)	41.9 a (±2.2)	40.7 a,b (±2.4)	37.0 b (±1.5)
Pathogen infestation, %	11.0 b (±1.51)	19.4 a (±1.60)	20.1 a (±1.75)	9.7 b (±1.25)	15.1 a (±1.23)	16.3 a (±1.32)

**Table 5:** The value of the LAI and IPAR indices and the content of chlorophyll depending on the foliar application of AgNPs treatments; urea solution with AgNPs (AgNPs + Urea), urea solution (Urea), water (Water). a, b—letters in the columns for LAI, IPAR, and Chlorophyll indicate a significant difference of their values at *p* < 0.05.

Treatment	Rape			Cucumber		
	LAI	IPAR	Chlorophyll	LAI	IPAR	Chlorophyll
2019						
AgNPs + Urea	2.37 a (±0.08)	71.4 a (±1.5)	632 a (±14.0)	3.57 a (±0.12)	81.2 a (±1.9)	461 (±8.8)
Urea	2.20 a,b (±0.10)	68.1 b (±1.7)	603 b (±12.6)	3.33 b (±0.09)	77.3 b (±1.6)	446 a,b (±9.9)
Water	2.11 b (±0.10)	67.8 b (±1.3)	605 b (±13.4)	3.26 b (±0.07)	77.3 b (±1.5)	439 b (±9.1)
2020						
AgNPs + Urea	4.12 a (±0.07)	85.6 a (±1.5)	567 a (±11.2)	4.63 a (±0.12)	88.6 a (±1.7)	516 a (±24.6)
Urea	3.82 b (±0.14)	80.9 b (±1.9)	558 a,b (±13.4)	4.67 a (±0.14)	87.1 a (±1.9)	509 a (±19.1)
Water	3.88 b (±0.07)	81.2 b (±1.5)	542 b (±9.9)	4.55 a (±0.15)	87.4 a (±1.3)	505 a (±14.7)
2021						
AgNPs + Urea	5.23 a (±0.11)	93.5 a (±3.7)	559 a (±7.6)	4.22 a (±0.06)	85.6 a (±1.3)	527 a (±16.4)
Urea	5.25 a (±0.10)	93.5 a (±2.4)	542 b (±8.7)	4.10 b (±0.06)	83.0 a,b (±1.5)	528 a (±12.8)
Water	5.16 a (±0.13)	92.6 a (±2.4)	542 b (±8.8)	4.06 b (±0.04)	82.4 b (±1.7)	519 a (±17.6)

## Data Availability

The data presented in this study are available in this article.
